# Co-expression of VP2, NS1 and NS2-Nt proteins by an MVA viral vector induces complete protection against bluetongue virus

**DOI:** 10.3389/fimmu.2024.1440407

**Published:** 2024-07-12

**Authors:** Luis Jiménez-Cabello, Sergio Utrilla-Trigo, Eva Calvo-Pinilla, Gema Lorenzo, Miguel Illescas-Amo, Julio Benavides, Sandra Moreno, Alejandro Marín-López, Aitor Nogales, Javier Ortego

**Affiliations:** ^1^ Centro de Investigación en Sanidad Animal (CISA), Instituto Nacional de Investigación y Tecnología Agraria y Alimentaria (INIA-CSIC), Madrid, Spain; ^2^ Instituto de Ganadería de Montaña, CSIC-Universidad de León, León, Spain; ^3^ Section of Infectious Diseases, Department of Internal Medicine, Yale University School of Medicine, New Haven, CT, United States

**Keywords:** bluetongue virus (BTV), vaccine, MVA, DIVA, multiserotype, sheep

## Abstract

**Introduction:**

Bluetongue (BT), caused by bluetongue virus (BTV), is an important arthropod-borne livestock disease listed by the World Organization for Animal Health. Live-attenuated and inactivated vaccines have permitted to control BT but they do not simultaneously protect against the myriad of BTV serotypes. Recently, we identified the highly conserved BTV nonstructural protein NS1 and the N-terminal region of NS2 as antigens capable of conferring multiserotype protection against BTV.

**Methods:**

Here, we designed Modified Vaccinia Ankara (MVA) viral vectors that expressed BTV-4 proteins VP2 or VP7 along with NS1 and NS2-Nt as well as MVAs that expressed proteins VP2, VP7 or NS1 and NS2-Nt.

**Results:**

Immunization of IFNAR(-/-) mice with two doses of MVA-NS1-2A-NS2-Nt protected mice from BTV-4M infection by the induction of an antigen-specific T cell immune response. Despite rMVA expressing VP7 alone were not protective in the IFNAR(-/-) mouse model, inclusion of VP7 in the vaccine formulation amplified the cell-mediated response induced by NS1 and NS2-Nt. Expression of VP2 elicited protective non-cross-reactive neutralizing antibodies (nAbs) in immunized animals and improved the protection observed in the MVA-NS1-2A-NS2-Nt immunized mice when these three BTV antigens were co-expressed. Moreover, vaccines candidates co-expressing VP2 or VP7 along with NS1 and NS2-Nt provided multiserotype protection. We assessed protective efficacy of both vaccine candidates in sheep against virulent challenge with BTV-4M.

**Discussion:**

Immunization with MVA-VP7-NS1-2A-NS2-Nt partially dumped viral replication and clinical disease whereas administration of MVA-VP2-NS1-2A-NS2-Nt promoted a complete protection, preventing viraemia and the pathology produced by BTV infection.

## 1 Introduction

Bluetongue (BT) is an important livestock disease transmitted by *Culicoides* biting midges (1). Traditionally, occurrence of BT has been enclosed to regions between approximately 40-50° N and 35° S (2) although worldwide spread has occurred, reaching northern latitudes and causing an economic impact estimated in more than 3 billion US dollars per year (3, 4). The causative agent of BT is Bluetongue virus (BTV), the archetypical member of the genus *Orbivirus* of the family *Sedoreoviridae* (5). This non-enveloped virus possesses an icosahedral capsid (~90 nm in diameter) divided in three concentric protein layers constituted by seven structural proteins, three of which (VP1, VP4 and VP6) are minor components in the viral particle compared to the major components VP2, VP5, VP3 and VP7 (6, 7). Apart from the seven aforementioned structural proteins, the ten doubled-stranded RNA (dsRNA) genomic segments located inside the inner core encode for five nonstructural proteins (NS1, NS2, NS3/NS3A, NS4 and NS5) (8, 9).

This disease affects wild and domestic ruminants, showing diverse mortality rates among ruminant species, with sheep and white-tailed deer (*Odocoileus virginianus*) as some of the most affected hosts (10). The lesions of bluetongue in sheep have been well described, and include oral erosions, ulcers, lameness and coronitis, weakness and depression, and facial edema (10–12). Cattle, goats and camelids usually show asymptomatic or sub-clinical disease (13). However, outbreaks of BTV-8 in Europe during 2006 caused clinical disease in cattle although data collected in the Netherlands BTV-8 epidemic showed that the mortality rate in cattle was 0 per 100 (14). BTV infection causes severe direct and indirect economic losses to livestock farmers due to high morbidity, stillbirths, abortions or fetal abnormalities, less birth weight, reduced milk yield and fertility rate and weight loss. Indirect losses are due to trade restrictions imposed on ruminant animal movement and vaccination, diagnosis and vector control costs (3, 15–17).

To date, more than 29 serotypes of BTV have been identified by phylogenetic studies, sequencing data and cross-neutralization assays (18). Due to the lack of therapeutic treatments (19), live-attenuated (LAV) and inactivated vaccines are the unique countermeasure to prevent and control of BT. However, alongside their low safety profile, these conventional vaccines lack DIVA (differentiating between infected and vaccinated animals) character, and they are serotype-specific as the protection induced is mainly mediated by VP2-specific nAbs. VP2, containing the majority of neutralizing epitopes and main determinant of virus serotype, recognizes the cell receptor and permits cell attachment during early stages of infection (20, 21), so that antibodies raised against this protein can block cell binding. Nonetheless, VP2 is highly variable among BTV serotypes, showing higher sequence variation in specific regions exposed to antigenic selection pressure (18). As a consequence, scarce cross-neutralizing relationships exist among BTV serotypes (22).

VP2 protein has been the primary antigen for vaccine development since the nAbs induced by VP2 are protective (23, 24). Nevertheless, highly conserved BTV proteins such as VP7, NS1 and NS2, are attractive targets to develop multiserotype responses. In this sense, the nonstructural protein NS1, the most expressed viral protein during the replicative cycle and almost identical among serotypes (25), contains T CD8+ epitopes and, more importantly, induces long-lasting protection against different BTV serotypes in the IFNAR(-/-) mouse model (26, 27). Similarly, the N-terminal half (amino acids 1 to 180) of the highly conserved nonstructural protein NS2 (25), NS2-Nt, also accommodates T-cell epitopes within its sequence (28). Individual expression of NS1 or co-expression of both NS1 and NS2-Nt induced a significant degree of protection against BTV in immunized sheep (27–29). Regarding VP7, several studies have pointed out the induction of multiserotype protective cell-mediated immune responses against BTV in the IFNAR(-/-) mouse model and natural hosts (30–32).

Nonetheless, no multivalent vaccine has been licensed yet for BT, but some experimental vaccine approaches have demonstrated multiserotype potential. For example, cocktails of VLPs (composed of VP2, VP5, VP7 and VP3) of different serotypes showed very good results in terms of broad protection in natural hosts (33). The subunit vaccine based on avian reovirus muNS-microspheres loaded with NS1, VP2 and VP7 also possessed multiserotype potential (34). A wide range of recombinant viral vectors have been designed for vaccine development against BTV, including poxviruses, adenoviruses or herpesviruses (23). Among poxviruses, the Modified Vaccinia Ankara (MVA) virus arose as an optimal viral vector due to its high safety profile, its capacity to accommodate large foreign DNA insert and its high immunogenicity *in vivo* (35). Several MVA-based vaccine candidates have been developed against human and veterinary viral diseases, with a high number of clinical and preclinical studies performed (35, 36). For BTV, homologous strategies based on recombinant MVA (rMVA) expressing NS1 and heterologous regimes combining rMVA and recombinant adenovirus ChAdOx1 co-expressing NS1 and NS2-Nt provided very promising results in terms of homologous and heterologous protection against BTV (26, 28, 29).

In this work, we studied the protective capacity of novel rMVA viral vectors co-expressing serotype 4 proteins VP2 or VP7 along with the immunogenic NS1 and NS2-Nt proteins of BTV. After confirming their immunogenicity and their ability to protect against BTV in the IFNAR(-/-) mouse model, we evaluated the protection conferred by a homologous prime-boost immunization strategy in sheep, one of the most affected natural hosts of BTV.

## 2 Materials and methods

### 2.1 Cells lines and viruses

Chicken embryo fibroblasts (DF-1) (ATCC, Cat. No. CRL-12203) were grown in Dulbecco’s Modified Eagle’s medium (DMEM) (Biowest, Nuaillé, France) supplemented with 2mM glutamine (Gibco, Waltham, MA, USA) and 10% fetal calf serum (FCS) (Gibco, Waltham, MA, USA). Green monkey kidney cells (Vero) (ATCC, Cat. No. CCL-81) were grown in DMEM supplemented with 2mM glutamine and 5% FCS.

BTV serotype 1 (ALG2006/01) (BTV-1), BTV serotype 4 Morocco strain (MOR2009/09) (BTV-4M), BTV serotype 4 (SPA2004/02) and BTV serotype 8 (BEL/2006) (BTV-8) were used in the experiments. BTV-4M strain is a reassortant strain between BTV-1 (segments 1, 4, 5, 7, 9, 10) and BTV-4 (segments 2, 3, 6, 8) isolated from sheep blood in KC insect cells (37, 38). Virus stocks and titrations were performed in Vero cells by standard methods previously described (39).

### 2.2 Generation of rMVA vaccine vectors

rMVAs containing genes encoding for BTV-4 VP2 or VP7 proteins placed in the F13L locus, rMVA co-expressing NS1 and NS2-Nt cloned as a single gene in the TK locus, and rMVAs simultaneously expressing VP2 or VP7 along with NS1 and NS2-Nt has been generated as previously has been described (40). For this purpose, transfer plasmids pMVA containing segment 2 or segment 7 from BTV-4 (SPA2004/02) were constructed. Shortly, VP2 and VP7 genes were amplified from previously generated plasmids pcDNA3-VP2 (39) and pSC11-VP7 (39) with primers specified in **Table 1**. The restriction sites EcoRI and BamHI were introduced at the 5’ and 3’ ends, respectively, of the PCR products. The DNA inserts were digested with EcoRI and BamHI restriction enzymes and were cloned into the MVA transfer plasmid pMVA-β-Gus (41) previously digested with the same restriction enzymes. Subsequently, plasmids pMVA-VP2 or pMVA-VP7 were transfected in DF-1 cells infected with MVAΔF13L that encodes dsRed marker instead of the native F13L ORF at a MOI of 1 using Lipofectamine™ 3000 Transfection Reagent (Invitrogen™, CA, USA), following the protocol facilitated by the manufacturer. Cell cultures were harvested at 48 hours post-infection (h.p.i.) and rMVAs were purified by plaque-picking and fluorescent selection in a Zeiss Axio fluorescence microscope (Zeiss, Oberkochen, Germany). Complementary, rMVAs were cloned at least five times by plaque assay for a greater purification.

**Table 1:** Primers designed to generate rMVA.

Primer	Sequence	Annealing Temperature
Fw-EcoRI-VP2^1^	5’-CGgaattcATGGAGGAGTTTGTCATTCC-3’	50°C
RS-BamHI-VP2^1^	5’-CGggatccCTAAACGTTGAGTAATTTCG-3’	50°C
Fw-EcoRI-VP7^2^	5’-CGgaattcATGGACACTATCGCTGCAAG-3’	60°C
RS-BamHI-VP7^2^	5’-CGggatccCTACACATAGGCGGCGCGTG-3’	60°C
Fw-XmaI-NS1^3^	5’-GAACAGTGACGGATCcccgggATGGAGCGTTTTTGAGAAAATAC-3’	62°C
Rs-XmaI-NS2-Nt^3^	5’-ACGCTCACAGAATTcccgggCTACGCCACGCTTTGAACTTG-3’	62°C

^1^Primers designed to generate pMVA-VP2. EcoRI and BamHI restriction sites are represented by lowercase letters.

^2^Primers designed to generate pMVA-VP7. EcoRI and BamHI restriction sites are represented by lowercase letters.

^3^Primers designed to generate pSC11-NS1-2A-NS2-Nt. XmaI restriction site is represented by lowercase letters.

Consecutively, MVA transfer plasmid pSC11 containing NS1-2A-NS2-Nt was used to generate rMVAs expressing VP2 or VP7 along with NS1 and NS2-Nt as well as a rMVA expressing just NS1 and NS2-Nt. For this purpose, NS1-2A-NS2-Nt was amplified from previously generated plasmid p1990 containing NS1-2A-NS2-Nt (28) with primers specified in **Table 1**. The restriction sites XmaI were introduced at the 5’ and 3’ ends of the PCR product. The DNA insert was digested with XmaI restriction enzyme and cloned into the MVA transfer plasmid pSC11 previously digested with the same restriction enzyme. Subsequently, plasmid pSC11-NS1-2A-NS2-Nt was transfected in DF-1 cells infected (MOI of 1) with the MVA-VP2 or MVA-VP7, generated in the prior step, or with wild-type MVA, using Lipofectamine™ 3000 Transfection Reagent (Invitrogen™, CA, USA), following the protocol facilitated by the manufacturer. This allows recombination of the transgene and the marker LacZ with the MVA genome in the native TK ORF. Cell cultures were harvested at 48 h.p.i. and selection of rMVAs was performed by plaque assay in presence of X-Gal. Complementary, rMVAs were cloned at least five times by plaque assay for a greater purification.

### 2.3 Indirect immunofluorescence microscopy

DF-1 cells were grown in glass coverslips and infected with MVA-VP2, MVA-VP7, MVA-NS1-2A-NS2-Nt, MVA-VP2-NS1-2A-NS2-Nt, MVA-VP7-NS1-2A-NS2-Nt at a MOI of 1, or non-infected. Twenty-four hours after infection, cell monolayers were fixed for 15 minutes with 4% paraformaldehyde. Fixed cells were blocked with 20% FBS-PBS-Saponine 0.2% (20% blocking solution) for 60 minutes at room temperature (RT). DF-1 cells were then incubated overnight at 4°C with a mouse hyperimmune serum against recombinant purified proteins BTV-4 VP2 (1:500) or VP7 (1:500), a serum from a mouse immunized with ChAdOx1-NS1 (27) (1:500) or the monoclonal antibody (mAb) 23H6 specific BTV NS2 protein (Eurofins INGENASA, Madrid, Spain) (1:500), diluted in PBS-FBS 20%. After three serial washing steps with PBS, DF-1 cells were incubated for 30 minutes at room temperature (RT) with Alexa Fluor 594 goat conjugated anti-mouse IgG (Invitrogen™, German Town, MD, USA) (1:500). Coverslips with infected DF-1 cells were washed three times with PBS and once with PBS-DAPI (1:10000), and visualized in a Zeiss Axio fluorescence microscope (Zeiss, Oberkochen, Germany). Adobe Photoshop CS5 Extended (Adobe Systems, CA, USA) was used afterwards for image editing.

### 2.4 Western blot analysis

DF-1 cells were infected with the previously generated rMVAs (MOI=0.1) or were mock infected. At 18 h.p.i., cells were harvested, washed in PBS containing protease inhibitor cocktail (Sigma-Aldrich, St. Louis, MO, USA), and lysed with RIPA Buffer (Santa Cruz Biotechnology, Dallas, TX, USA). Then, extracts were sonicated for 2 minutes and proteins were resolved in 12% SDS-PAGE and blotted onto a nitrocellulose membrane. After a blocking step with 5% low fat dry milk in TBS Tween-20 (TBST) (blocking buffer) membranes were incubated with α-BTV NS2 mAb 23H6 (1:500) in TBST-Milk 5% overnight at 4°C. Bound antibody was detected with horseradish peroxidase-conjugated rabbit anti-mouse antibody (Sigma-Aldrich, San Louis, MO, USA) diluted in TBST-Milk 5% (1:10000) and the ECL detection system (AmershamTM Pharmacia Biotech, Buckinghamshire, UK).

### 2.5 Mice and sheep

Type I interferon receptor defective mice [IFNAR (-/-)] on a 129 Sv/Ev background and sheep (Ovis aries “Churra” breed) were used for the studies. All mice and sheep used were matched for age (8 weeks and 4 months, respectively). Mice and sheep were housed under pathogen-free conditions and allowed to acclimatize to the biosafety level 3 (BSL3) animal facilities at the Animal Health Research Center (CISA-INIA, CSIC), Madrid, before use.

### 2.6 Mice immunization and challenge

Two different immunization strategies were evaluated in mice. First, a set of five groups of mice (n=5) were intraperitoneally immunized with a single dose of 1x10^7^ PFU per mouse of rMVAs (MVA-VP2, MVA-VP7, MVA-NS1-2A-NS2-Nt, MVA-VP2-NS1-2A-NS2-Nt or MVA-VP7-NS1-2A-NS2-Nt). A second set of five groups of mice (n=5) were intraperitoneally immunized following a homologous prime-boost regime consisting of two doses of 1x10^7^ PFU per mouse of MVA-VP2, MVA-VP7, MVA-NS1-2A-NS2-Nt, MVA-VP2-NS1-2A-NS2-Nt or MVA-VP7-NS1-2A-NS2-Nt, administered four weeks apart. Additionally, a group of mice (n=5) was subjected to the prime-boost immunization strategy with the MVA-VP2-NS1-2A-NS2-Nt or MVA-VP7-NS1-2A-NS2-Nt for the multiserotype protection experiment. A group of mice (n=5) was left untreated (control) for each experiment.

Animals were subcutaneously challenged with a lethal dose (10 PFU) of BTV-4M four weeks after immunization in the case of those animals given a single dose of rMVA. Animals subjected to the homologous prime-boost MVA/MVA strategy were challenged with a lethal dose of BTV-4M (10 PFU) at three w.p.b. For the multiserotype protection experiment, animals were subcutaneously challenged with a lethal dose (100 PFU) of BTV-1 at three w.p.b. In all cases, submandibular blood collection was carried out in mice after virus challenge at 3, 5, 7, 10 and 14 d.p.i. for the analysis of viremia.

### 2.7 Sheep immunization and challenge

A total of 12 naive healthy sheep (Spanish “Churra” sheep breed), aged 6 months, were acclimated for seven days at the BSL3 animal facility of the Animal Health Research Center (CISA-INIA, CSIC) before starting the experiment. All sheep involved in the experiment were negative to BTV antibodies by ELISA. Briefly, two groups of sheep (n=4) were intramuscularly immunized following a homologous primer-boost strategy consisting of two doses of 10^8^ PFU of MVA-VP2-NS1-2A-NS2-Nt or MVA-VP7-NS1-2A-NS2-Nt, administered four weeks apart. A group of sheep was left untreated (control). Pre-challenge blood samples were collected from all animals. Non-immunized and immunized sheep were subcutaneously challenged with a dose of 10^5^ PFU of BTV-4M at three w.p.b. After virus challenge, blood collection for virological analyses was conducted by specialized veterinary personal at 0, 3, 5, 7, 10, 12, 14, and 18 d.p.i. Rectal temperatures measurements were conducted every day from 7 days prior to challenge until 18 d.p.i. At day 18 post-infection all sheep were euthanized.

### 2.8 Viraemia and RNAemia analysis by plaque assay and RT-qPCR

Blood samples were collected at 3, 5, 7, 10 and 14 d.p.i from the submandibular plexus of mice and at 0, 3, 5, 7, 10, 12, 14, and 18 d.p.i. from sheep with EDTA as anti-coagulant.

For the analysis of RNAemia by RT-qPCR, RNA was extracted from 50 µL of blood using TRIzol Reagent (Sigma Aldrich, St. Louis, MO, USA) following the protocol established by the manufacturer. RNAemia was analyzed in duplicate by real-time RT-qPCR specific for BTV segment 5 (encoding for NS1). The real-time RT-qPCR specific for BTV segment 5 was performed using primers and probe described by Toussaint et al. (42). Only Ct values lower than 38 were considered indicative of RNAemia (positive), according to the cut-off established by Toussaint et al. (42). Mice and sheep blood containing different concentrations of virus were titrated and used as internal standards of the experiment (26).

For the analysis of viraemia by plaque assay, 50 µL of sheep blood were diluted in PBS1X and centrifuged at 3000 rpm for 10 minutes. Thereafter, supernatant was removed, and pellet was lysed in 450 µL of sterile water for 2 minutes. Cell lysis was stopped by adding 50 µL of PBS10X. Then, different volumes of samples were inoculated into 12-well plates containing semi-confluent monolayers of Vero cells. Following incubation for 1 h, an agar overlay (DMEM-10%-FBS-0.4%-Noble Agar, Becton Dickinson, MD, USA) was added and plates were incubated for 5 days at 37°C in 5% CO_2_. Plaques were fixed with 10% formaldehyde and visualized with 2% crystal violet-PBS.

### 2.9 Blood measurements

A multiparameter autohematology analyzer (BC-5300 Vet; Mindray, China) was used to determine the total and differential cell counts in sheep blood for each group and collected into EDTA tubes.

### 2.10 Ex vivo flow cytometric analysis

To evaluate the immunogenicity of the rMVAs in mice, a set of five groups of IFNAR(-/-) mice (n=4) was subjected to a homologous prime-boost regimen (MVA/MVA-VP2, MVA/MVA-VP7, MVA/MVA-NS1-2A-NS2-Nt, MVA/MVA-VP2-NS1-2A-NS2-Nt or MVA/MVA-VP7-NS1-2A-NS2-Nt). rMVAs were inoculated intraperitoneally in a four-week interval. For this study, one group of mice (n=4) was left untreated (control). All animals were euthanized at 15 days post-boost, and their spleens were harvested for analysis by ICS.

A total of 10^6^ splenocytes per well were stimulated with 5 μg/ml of VP2 (from BTV-4) protein, 5 μg/ml of VP7 protein, 5 μg/ml of NS1-152 peptide (9-mer peptide GQIVNPTFI), 5 μg/ml of the NS2-Nt protein, concanavalin A (ConA) as a nonspecific stimulus (4 μg/ml) for 5h (18h in the case of NS2-Nt protein) or left untreated in RPMI 1640 medium supplemented with 10% FCS. Six hours before the assay, CD107a/LAMP-1-FITC antibody at 1:10 dilution (Miltenyi, Biotec, Bergisch Gladbach, Germany) and brefeldin A (5 µg/ml) were added. After stimulation, cells were washed with PBS-1%-FBS, stained for the surface markers, fixed with PBS-1%-FBS-1%-Saponine-4%-PFA, permeabilized with PBS-1%-FBS-1%-Saponine, and stained intracellularly using the fluorochrome conjugated antibody IFN-γ–PE (Miltenyi Biotec, Bergisch Gladbach, Germany). Fluorochrome conjugated antibodies CD8-PerCP-Vio700, CD62L-APC and CD127-FITC (Miltenyi Biotec, Bergisch Gladbach, Germany) were used for the analysis of extracellular receptor molecules. Data were acquired by FACS analysis on a FACSCalibur platform (Becton Dickinson, Franklin Lakes, NJ, USA). Analyses of the data were performed using FlowJo software version x0.7 (Tree Star, Ashland, OR, USA). The number of lymphocyte-gated events was 5x10^5^. Lymphocytes were initially gated on the basis of their forward and side scatter properties. Then, CD8+ lymphocytes expressing IFN-γ or CD107a were selected for the analysis. Gating strategies used to identify CD8+ T-cell populations are showed in the **Supplementary Figure 1**.

### 2.11 Plaque reduction neutralization test

Two-fold dilutions (from 1:5) of heat inactivated mice or sheep sera (56°C for 30 minutes) were incubated with 100 PFU of BTV-4, BTV-1 or BTV-8, for 1h at 37°C. Then, samples were inoculated into 12-well plates containing semi-confluent monolayers of Vero cells. Following incubation for 1h, an agar overlay [DMEM-10%-FBS-0.4%-Noble Agar (Becton Dickinson, MD, USA)] was added and plates were incubated for 5 days at 37°C in 5% CO_2_. Plaques were fixed with 10% formaldehyde and visualized with 2% crystal violet-PBS. PRNT_50_ titer was calculated as the highest dilution of serum that neutralized 50% of the control virus input.

### 2.12 Detection of antibodies specific of VP7 by ELISA

MaxiSorp plates (Nunc) (Thermo Fisher Scientific, NY, USA) were coated with VP7 (50 ng per well) purified baculovirus expressed protein in PBS and incubated overnight at 4°C. Plates were saturated with blocking buffer (PBS-0.05%-Tween 20-5% skim milk). Individual sheep sera diluted in blocking buffer (1:200) were added and incubated for 2h at 37°C. After three washes in PBS-0.05% Tween 20, plates were incubated for 1h at 37°C with an anti-sheep-HRP secondary antibody (Sigma-Aldrich, San Louis, MO, USA) (1:7500) in blocking buffer. Finally, after three washes in PBS-0.05% Tween 20, the reaction was developed with 50 µL of TMB (Thermo Fisher Scientific, MD, USA) and stopped by adding 50 µL of 3 N H_2_SO^4^ (Merck, Darmstadt, Germany). Results were expressed as optical densities (ODs) measured at 450 nm.

### 2.13 Statistical analysis

Data were analyzed using GraphPad Prism version 8.0.1 (GraphPad Software, San Diego, CA, USA). Survival curves for each immunized mice group were compared to those of non-immunized mice in search of statistical differences using Log-rank test. Comparisons of mean responses between groups in the RNAemia analysis for mice group were performed using Mann-Whitney non-parametric test. Comparisons of mean responses between groups for the ICS, PRNT_50_ and ELISA assays as well as data on rectal temperature, viraemia, RNAemia and hematologic values were conducted by two-way ANOVA with a *post hoc* Tukey test for multiple comparisons. A p-value lower than 0.05 was considered significant in all cases.

### 2.14 Ethics statement

Animal experimental protocols were approved by the Ethical Review Committee at the INIA-CISA and Comunidad de Madrid (Permit number: PROEX 060.7/21) in strict accordance with EU guidelines 2010/63/UE about protection of animals used for experimentation, and other scientific purposes and Spanish Animal Welfare Act 32/2007.

## 3 Results

### 3.1 Evaluation of BTV-4 VP2, VP7, NS1 and NS2-Nt expression from rMVAs

Lately, we observed the induction of strong antigen-specific humoral and cell-mediated immune responses in animals immunized with rMVA after cloning of heterologous antigens in the F13L and TK loci of the MVA genome (27–29). Thus, we generated rMVAs that individually express BTV genes that encode for BTV-4 proteins VP2 or VP7 cloned in the F13L locus (MVA-VP2 or MVA-VP7) and a rMVA that co-expresses BTV genes that encode for NS1 and NS2-Nt cloned in the TK locus as a single fused gene (MVA-NS1-2A-NS2-Nt). Additionally, we also designed rMVAs that co-expressed VP2 or VP7 along with NS1 and NS2-Nt (MVA-VP2-NS1-2A-NS2-Nt or MVA-VP7-NS1-2A-NS2-Nt).

**Figure 1:**
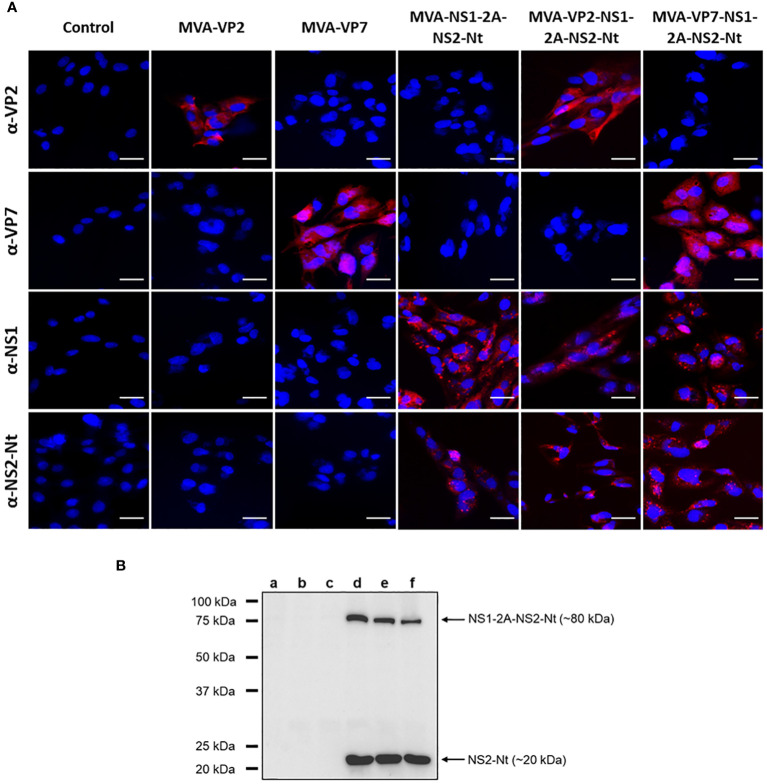
Expression analysis of heterologous BTV proteins by rMVA. **(A)** Indirect immunofluorescence of DF-1 cells infected (MOI = 1) with MVA-VP2, MVA-VP7, MVA-NS1-2A-NS2-Nt, MVA-VP2-NS1-2A-NS2-Nt, MVA-VP7-NS1-2A-NS2-Nt or non-infected (control). VP2 and VP7 protein were detected using a mouse polyclonal hyperimmune serum against VP2 or VP7, respectively. NS1 protein was detected using a mouse polyclonal hyperimmune serum against ChAdOx1-NS1. NS2-Nt was detected using MAb 23H6 α-NS2. Nuclei were stained with DAPI. Scale bars 20 μm. **(B)** Immunoblot analysis of non-infected DF-1 cells (lane a) or infected with MVA-VP2 (lane b), MVA-VP7 (lane c), MVA-NS1-2A-NS2-Nt (lane d), MVA-VP2-NS1-2A-NS2-Nt (lane e) or MVA-VP7-NS1-2A-NS2-Nt (lane f) at 18 h.p.i. using a MAb 23H6 α-NS2. Numbers indicate relative molecular mass in Kilodaltons (kDa).

The proper expression of these heterologous BTV antigens cloned in the rMVAs was analyzed by indirect immunofluorescence assay (IFA). The characteristic spotted pattern of NS1 was observed after infection of DF-1 cells with MVA-NS1-2A-NS2-Nt, MVA-VP2-NS1-2A-NS2-Nt or MVA-VP7-NS1-2A-NS2-Nt (**Figure 1A**). Likewise, we noted a specific signal corresponding with the expression of NS2-Nt in DF-1 cells infected with these rMVAs (**Figure 1A**). The expression of BTV proteins VP2 and VP7 was also revealed in DF-1 cells infected with the correspondent rMVAs expressing these BTV antigens (**Figure 1A**). Non-infected cells did not show any evidence of a specific signal of VP2, VP7, NS1 or NS2-Nt in any case.

To maximize the cloning capacity of the MVA viral vector, we cloned the genes that encode for the proteins NS1 and NS2-Nt as a single gene with the foot-and-mouth disease virus (FMDV) 2A “ribosomal skipping” linker (2A) included in the site of fusion, which will eventually lead to individual expression of these BTV antigens in infected cells. However, 2A linker do not usually show a 100% “self-cleaving” efficiency (43). To confirm the separate expression of these proteins, we conducted an immunoblotting assay marking the protein NS2-Nt, observing the individual expression of NS2-Nt (~20 kDa) in DF-1 cell extracts infected with MVA-NS1-2A-NS2-Nt, MVA-VP2-NS1-2A-NS2-Nt or MVA-VP7-NS1-2A-NS2-Nt (**Figure 1B**, lanes d, e and f). Nonetheless, the monoclonal Ab 23H6 specific of NS2-Nt also permitted to detect the fused NS1-2A-NS2-Nt insert (~80 kDa), as previously observed (28), which indicates the individual expression of both NS1 and NS2-Nt BTV proteins as well as the expression of a polyprotein formed by NS1 and NS2-Nt.

Altogether, these results confirm the correct expression of the heterologous BTV antigens cloned in the rMVAs to be used for pre-clinical assays in IFNAR(-/-) mice.

### 3.2 Immunogenicity of rMVA vaccine candidates in IFNAR(-/-) mice

**Figure 2:**
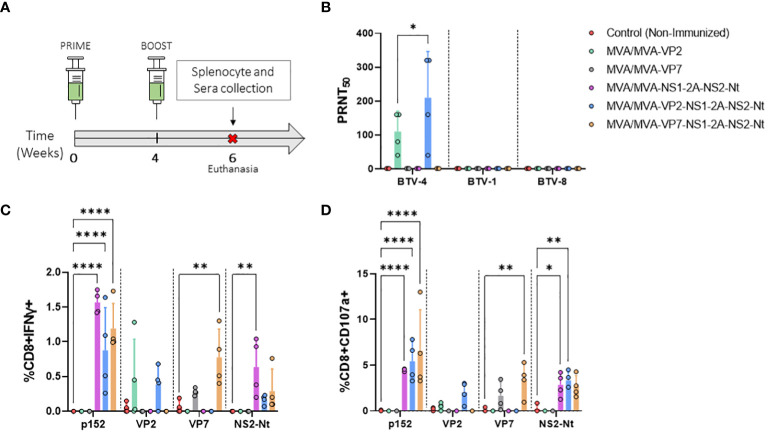
Immunogenicity of vaccine candidates in IFNAR(-/-) mice. **(A)** Groups of IFNAR(-/-) mice (n=4) were immunized following a homologous prime-boost regimen consisting of two doses of MVA-VP2, MVA-VP7, MVA-NS1-2A-NS2-Nt, MVA-VP2-NS1-2A-NS2-Nt, MVA-VP7-NS1-2A-NS2-Nt in a four-week interval. A group was non-immunized (control). Splenocyte and blood collection were performed two w.p.b. **(B)** nAbs titers against BTV-4, BTV-1 or BTV-8 in immunized animals by plaque reduction neutralization assay. Points represent individual values for each mouse, bars represent the mean values of each group and error bars represent SD. Statistical differences between groups were analyzed using two-way ANOVA (*post hoc* Tukey test for multiple comparisons). **(C, D)** Percentage of CD8+IFN-γ+ T cells **(C)** and CD8+CD107a+ T cells **(D)** after restimulation with peptide 152 (NS1) or proteins VP2, VP7, or NS2-Nt. Points represent individual values for each mouse, bars represent the mean values of each group and error bars represent SD. Asterisks denote significant differences between immunized and control mice [two-way ANOVA (*post hoc* Tukey test for multiple comparisons)]. *P value <0.05, **P value <0.002, ****P value <0.0001.

The heterologous BTV proteins cloned in the rMVAs have been previously described as highly immunogenic, with protein VP2 of BTV as the major inductor of nAbs and proteins VP7, NS1 and NS2-Nt able to stimulate potent cytotoxic T lymphocytes (CTLs) responses. Prior to evaluate the protective capacity of our vaccine candidates, we assessed their ability to induce an immunogenic response. To that end, groups of IFNAR(-/-) mice (n=4) were intraperitoneally immunized with rMVAs following a prime-boost strategy. Two weeks after the boost dose, mice were euthanized, and their spleens and blood were harvested (**Figure 2A**).

First, we assessed whether our rMVA viral vectors expressing VP2 of serotype 4 were capable of eliciting homologous and heterologous nAbs. To do so, PRNT_50_ titers against BTV-1, BTV-4 and BTV-8 were determined. Immunization with two doses of MVA-VP2 or MVA-VP2-NS1-2A-NS2-Nt successfully induced high nAbs titers against the homologous BTV-4 (**Figure 2B**). nAbs titers against BTV-4 were very similar after prime-boost immunization with MVA-VP2-NS1-2A-NS2-Nt compared to the MVA-VP2 immunization. As could be expected, since the identity among VP2 proteins of serotypes 1, 4 and 8 used in this work analyzed with UniProt was between 40.76% and 52.92%, no cross-neutralizing Abs were detected against the heterologous BTV-1 or BTV-8 (**Figure 2B**).

Thereafter, to analyze the cellular immune response elicited by the rMVA, we measured IFN-γ production as well as CD107a cytotoxic expression marker in CD8+ T cells by Intracellular Cytokine Staining (ICS) after restimulation of splenocytes from immunized and non-immunized mice with the NS1 immunodominant peptide p152 (9-mer peptide GQIVNPTFI) or the purified recombinant proteins VP2, VP7 or NS2-Nt (**Figures 2C, D**). Significantly higher levels of CD8+IFN-γ+ as well as CD8+CD107a+ T cells were observed in comparison with the non-immunized control group upon restimulation with p152 of splenocytes from mice immunized with MVA-NS1-2A-NS2-Nt, MVA-VP2-NS1-2A-NS2-Nt or MVA-VP7-NS1-2A-NS2-Nt. Similarly, higher levels of CD8+IFN-γ+ and CD8+CD107a+ T cells were also recorded for these three immunization groups compared to the control group after restimulation with the recombinant protein NS2-Nt. A VP7-specific cytotoxic response was observed after restimulation with the recombinant protein VP7 of splenocytes from mice immunized with rMVA expressing this BTV antigen alone or combined with NS1 and NS2-Nt. Non-immunized animals displayed an almost unperceivable response to this stimulus. Interestingly, after stimulating splenocytes from animals immunized with MVA-VP2 or MVA-VP2-NS1-2A-NS2-Nt with the recombinant VP2 protein, a detectable increase of CD8+IFN-γ+ as well as CD8+CD107a+ T cells was observed compared to the control group.

### 3.3 Evaluation of the protection conferred by the rMVAs against BTV-4M in IFNAR(-/-) mice

**Figure 3:**
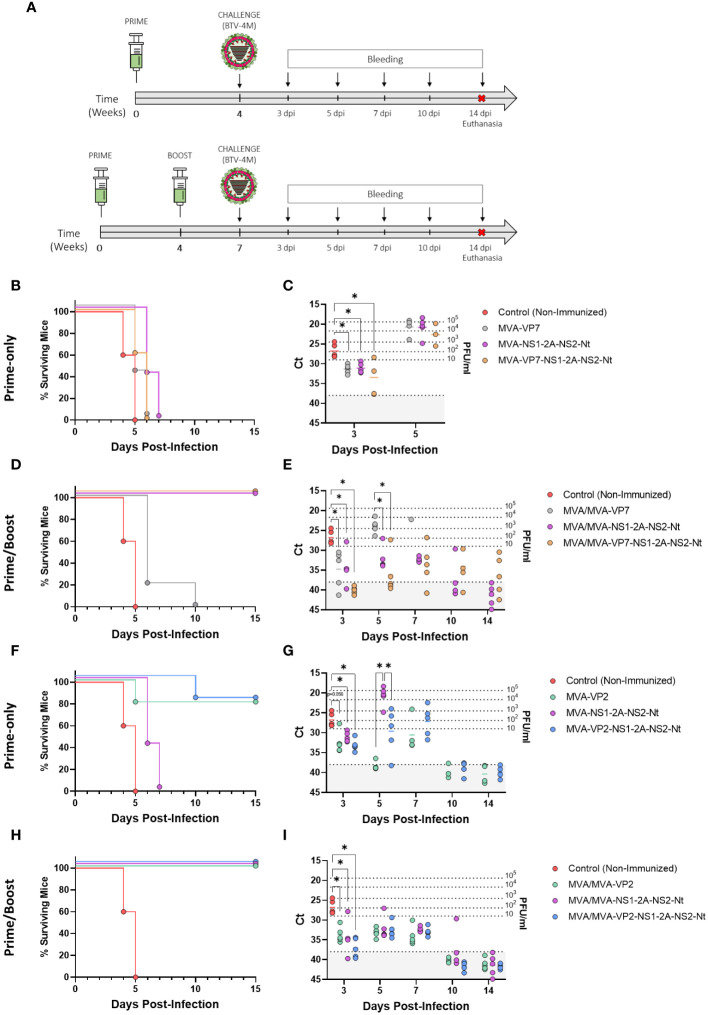
Protection of immunized IFNAR(-/-) mice against a lethal challenge with BTV-4M. **(A)** Groups of IFNAR(-/-) mice (n=5) were immunized with **(B, C, F, G)** a single dose of MVA-VP2, MVA-VP7, MVA-NS1-2A-NS2-Nt, MVA-VP2-NS1-2A-NS2-Nt, MVA-VP7-NS1-2A-NS2-Nt or **(D, E, H, I)** following a homologous prime-boost regimen consisting of two doses of these rMVAs. A group was left untreated (Control). Immunized and non-immunized mice were challenged with a lethal dose of BTV-4M. **(B, D, F, H)** Survival rates after infection. Curves were found statistically significant compared to non-immunized survival curve as calculated by Log-rank test (p-value < 0.05). **(C, E, G, I)** RNAemia analyzed by RT-qPCR of non-immunized and immunized IFNAR(-/-) mice after viral challenge. Expression of mRNA of segment 5 (encoding NS1 protein) was quantified at 3, 5, 7, 10 and 14 d.p.i. Results were expressed as Ct (left y-axis) and PFU/ml equivalents (right y-axis and dotted horizontal lines). The real-time RT-qPCR specific for BTV segment 5 was performed as described by Toussaint et al. (42) and mouse blood containing different concentrations of virus were titrated and used as standards (26, 27). Cut-off Ct ≥ 38 (dotted grey line). Points represent individual Ct for each mouse and lines of the corresponding color represent the mean Ct value of each group. Differences between groups were calculated by multiple t test analysis using the Sidak–Bonferroni method. *p-value <0.05.

Previous studies have pointed out the protective capacity of the nonstructural proteins NS1 and NS2-Nt of BTV (27–29) as well as the protection induced by the BTV structural proteins VP2 (33) and VP7 (30) against homologous BTV serotypes. Considering the results on immunogenicity of our MVA-based vaccines candidates, we decided to test their protective potential against a homologous BTV challenge. To that end, groups of IFNAR(-/-) mice (n=5) were intraperitoneally immunized with a single dose of 1x10^7^ PFU of rMVAs (MVA-VP2, MVA-VP7, MVA-NS1-2A-NS2-Nt, MVA-VP2-NS1-2A-NS2-Nt or MVA-VP7-NS1-2A-NS2-Nt) or following a homologous prime-boost immunization regimen in a four-week interval (**Figure 3A**). Four (prime-only) or three (prime-boost) weeks after the last immunization, mice were subcutaneously challenged with a lethal dose (10 PFU) of BTV-4M. Survival and RNAemia were subsequently analyzed.

All control mice succumbed to BTV infection between days 4 and 5 post-infection. Mice receiving a single dose of rMVA expressing VP7, individually or in combination with NS1 and NS2-Nt, as well as those mice immunized with a single dose of MVA-NS1-2A-NS2-Nt, experimented a delay in the day of death compared to the control group but all of them died after 7 days post-infection (d.p.i.) (**Figure 3B**). Importantly, these immunized mice showed significantly lower RNA levels in blood at 3 d.p.i. (MVA-VP7 Ct value mean = 31.36; MVA-NS1-2A-NS2-Nt Ct value mean = 31.09; MVA-VP7-NS1-2A-NS2-Nt Ct value mean = 33.468) compared to the non-immunized control group (Ct value mean = 26.788) (**Figure 3C**). Indeed, two out of five mice immunized with a single dose of MVA-VP7-NS1-2A-NS2-Nt were nearly aRNAemic at this day, with Ct values almost reaching 38. Mice immunized with two doses of MVA-VP7 died after 6 d.p.i., except for one mouse surviving until day 10 post-infection, and they displayed significantly lower RNAemia levels (MVA/MVA-VP7 Ct value mean = 31.36) compared to the control group at day 3 post-infection (**Figures 3D, E**). In contrast, immunization with two doses of rMVA expressing NS1 and NS2-Nt, alone or combined with VP7, prevented animals from death (**Figure 3D**) although RNAemia was detectable throughout the experiment (**Figure 3E**). Again, RNAemia levels were significantly lower at day 3 post-infection (MVA/MVA-NS1-2A-NS2-Nt Ct value mean = 34.384; MVA/MVA-VP7-NS1-2A-NS2-Nt Ct value mean = 40.086) compared to the control group. Only animals immunized with two doses of MVA-VP7-NS1-2A-NS2-Nt did not show detectable RNAemia (Ct value ≥ 38) at 3 d.p.i. At 5 d.p.i., this MVA/MVA-VP7-NS1-2A-NS2-Nt immunization group showed lower levels of RNA in blood (MVA/MVA-VP7-NS1-2A-NS2-Nt Ct value mean = 36.182) than those of the MVA/MVA-NS1-2A-NS2-Nt immunization group (MVA/MVA-NS1-2A-NS2-Nt Ct value mean = 32.076), with four out of five immunized mice displaying the aRNAemic status (Ct value ≥ 38). Besides, immunization with two doses of MVA-VP7-NS1-2A-NS2-Nt led to significantly lower RNAemia at day 5 post-infection compared to animals immunized with MVA/MVA-VP7 (Ct value mean = 23.906). Overall, these data indicate that whereas expression of VP7 by rMVAs is not enough to confer full protection against a homologous challenge in the IFNAR(-/-) mouse model, there is a synergistic effect of VP7, NS1 and NS2-Nt able to cushion the raise of viraemia in immunized animals.

Regarding the protein VP2, we noticed a high degree of protection after just a single dose of rMVA expressing VP2 alone or in combination with NS1 and NS2-Nt. We observed 80% of survival rates for these two immunization groups although mortality was delayed in the MVA-VP2-NS1-2A-NS2-Nt immunization group compared to the MVA-VP2 immunized mice (**Figure 3F**). Besides, these animals displayed lower RNA levels at 3 d.p.i. (MVA-VP2 Ct value mean = 32.462; MVA-VP2-NS1-2A-NS2-Nt Ct value mean = 33.126) compared to the control group and showed an aRNAemic status (Ct value ≥ 38) from day 10 post infection (**Figure 3G**). Moreover, during the viral RNA peak at 5 d.p.i., a single dose of MVA-VP2-NS1-2A-NS2-Nt elicited a significant reduction of RNAemia (MVA/MVA-VP2-NS1-2A-NS2-Nt Ct value mean = 29.628) compared to the MVA-NS1-2A-NS2-Nt immunization group (MVA-NS1-2A-NS2-Nt Ct value mean = 20.8). Not surprisingly, immunization with two doses of MVA-VP2 or MVA-VP2-NS1-2A-NS2-Nt completely protected mice from BTV-4M infection (**Figure 3H**), with significantly lower RNA levels at 3 d.p.i. (MVA/MVA-VP2 Ct value mean = 33.706; MVA/MVA-VP2-NS1-2A-NS2-Nt Ct value mean = 37.042) compared to the non-immunized animals and with nearly undetectable RNAemia at 5 and 7 d.p.i., before viral clearance (**Figure 3I**). Altogether, these results indicate that the rMVAs expressing either VP2, NS1-2A-NS2-Nt or a combination of these three antigens, are efficacious in protection against a homologous BTV infection. Besides, no interference of any kind was observed in terms of protection when VP2, NS1 and NS2-Nt were co-expressed by the same rMVA.

### 3.4 rMVAs co-expressing BTV-4 VP2 or VP7 and NS1-2A-NS2-Nt protect against heterologous BTV-1 challenge in IFNAR(-/-) mice

**Figure 4:**
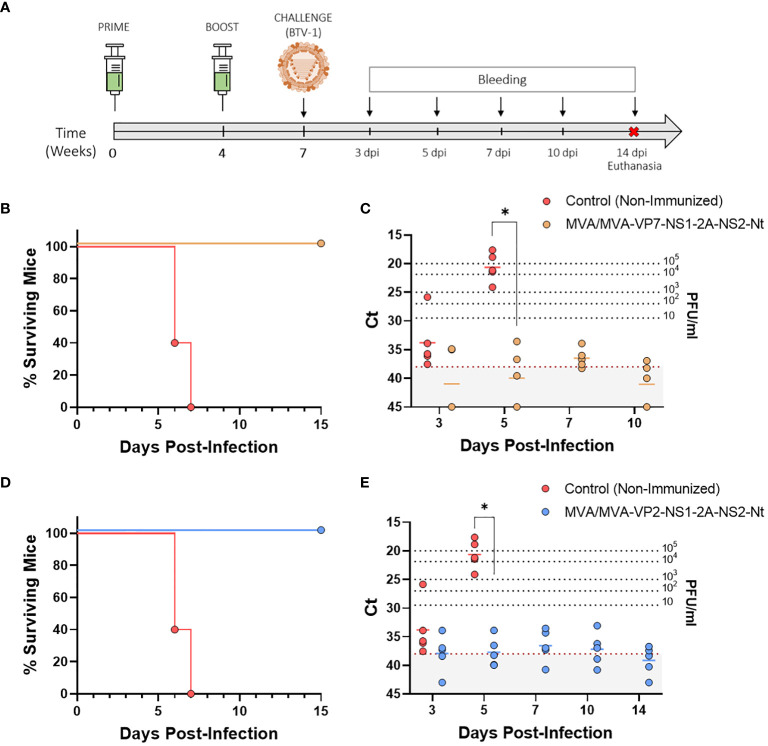
Protection of immunized IFNAR(-/-) mice against a lethal challenge with BTV-1. **(A)** A group of IFNAR(-/-) mice (n=5) were immunized following a homologous prime-boost regimen consisting of two doses of MVA-VP2-NS1-2A-NS2-Nt or MVA-VP7-NS1-2A-NS2-Nt. A group was left untreated (Control). Immunized and non-immunized mice were challenged with a lethal dose of BTV-1. **(B, D)** Survival rates after infection. Curve was found statistically significant compared to non-immunized survival curve as calculated by Log-rank test (p-value < 0.05). **(C, E)** Viremia analyzed by RT-qPCR of non-immunized and immunized IFNAR(-/-) mice after viral challenge. Expression of mRNA of segment 5 (encoding NS1 protein) was quantified at 3, 5, 7, 10 and 14 d.p.i. Results were expressed as Ct (left y-axis) and PFU/ml equivalents (right y-axis and dotted horizontal lines). The real-time RT-qPCR specific for BTV segment 5 was performed as described by Toussaint et al. (42) and mouse blood containing different concentrations of virus were titrated and used as standards (26, 27). Cut-off Ct ≥ 38 (dotted grey line). Points represent individual Ct for each mouse and lines of the corresponding color represent the mean Ct value of each group. Differences between groups were calculated by multiple t test analysis using the Sidak–Bonferroni method. *p-value <0.05.

Lately, we described the capacity of the protein NS1 alone or combined with NS2-Nt to induce durable cross-protective immune responses in the IFNAR(-/-) mouse model against different BTV serotypes (28). To assess the multiserotype potential of MVA-VP7-NS1-2A-NS2-Nt and MVA-VP2-NS1-2A-NS2-Nt, the vaccine candidates that generated better protection against a homologous challenge with BTV-4 in the prior step, we immunized IFNAR(-/-) mice (n=5) with two doses of 1x10^7^ PFU of MVA-VP2-NS1-2A-NS2-Nt or MVA-VP7-NS1-2A-NS2-Nt, in a four-week interval. Three weeks post-boost (w.p.b.), animals were subcutaneously challenged with a lethal dose (100 PFU) of the heterologous BTV-1, and survival and RNAemia were analyzed (**Figure 4A**).

All mice belonging to the non-immunized control group died by day 7 post-infection, showing peak RNAemia levels at day 5 post-infection (Ct value mean = 20.646) (**Figures 4B, C**). In contrast, immunization with two doses of either MVA-VP7-NS1-2A-NS2-Nt or MVA-VP2-NS1-2A-NS2-Nt completely protected mice from BTV-1 infection. Both immunized groups displayed a 100% survival rate and significantly lower RNA levels compared to the control group at 5 d.p.i. (MVA/MVA-VP2-NS1-2A-NS2-Nt Ct value mean = 37.7; MVA/MVA-VP7-NS1-2A-NS2-Nt Ct value mean = 39.904). Thereafter, immunized animals reached the aRNAemic status or nearly aRNAemic Ct values the remaining time points evaluated (**Figures 4B–E**). These data confirm that the MVA-vectored vaccine candidate co-expressing NS1 and NS2-Nt along with VP2 or VP7 can elicit amultiserotype protective response against BTV.

### 3.5 Protective capacity of rMVAs in sheep against BTV

**Figure 5:**
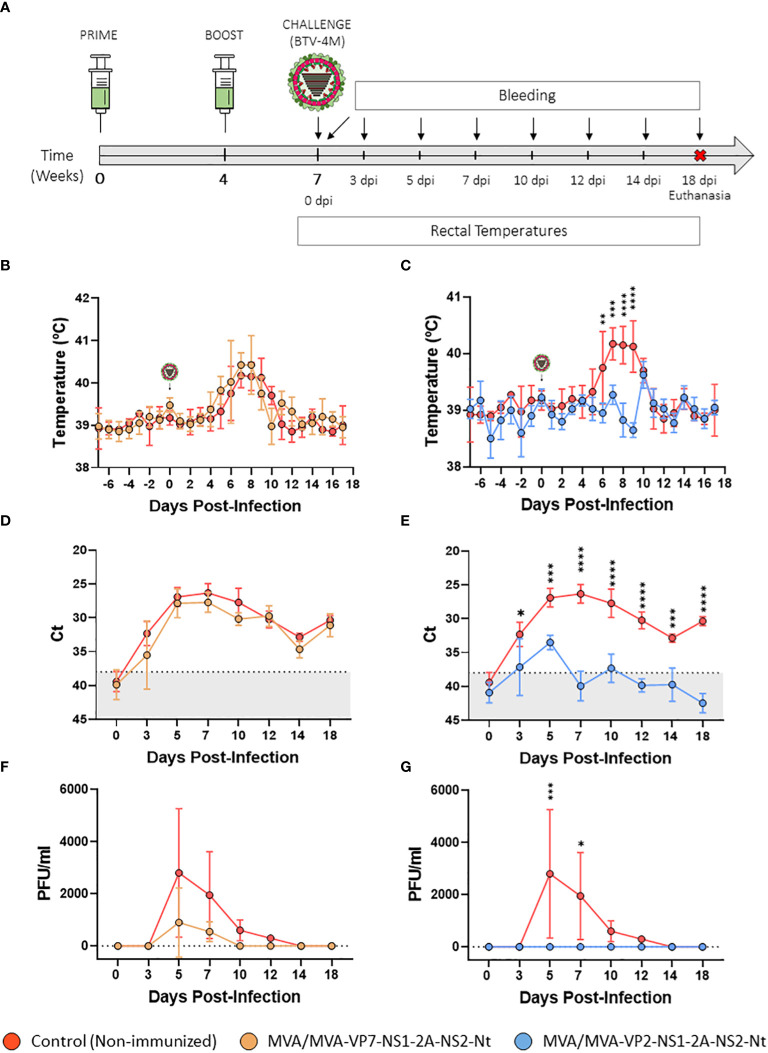
Protection of immunized sheep against a virulent challenge with BTV-4M. **(A)** Groups of sheep (n=4) were immunized following a homologous prime-boost regimen consisting of two doses of MVA-VP2-NS1-2A-NS2-Nt or MVA-VP7-NS1-2A-NS2-Nt. A group was left untreated (Control). Immunized and non-immunized sheep were challenged with BTV-4M. **(B, C)** Rectal temperatures recorded before and after challenge. The day of challenge (0 d.p.i.) is indicated. Points represent mean rectal temperature value for each group and error bars represent SD. **(D, E)** RNAemia analyzed by RT-qPCR of non-immunized and immunized sheep at 0, 3, 5, 7, 10, 14 and 18 d.p.i. Presence of virus in blood and expression of mRNA of segment 5 (encoding NS1 protein) was quantified at 0, 3, 5, 7, 10, 14 and 18 d.p.i. Results were expressed as Ct (left y-axis) and PFU/ml equivalents (right y-axis and dotted horizontal lines). The real-time RT-qPCR specific for BTV segment 5 was performed as described by Toussaint et al. (42). Cut-off Ct ≥ 38 (dotted grey line). Points represent mean Ct for each group and error bars represent SD. **(F, G)** Titers of BTV-4M recovered in blood of sheep after viral inoculation. Points represent mean PFU/ml value for each group and error bars represent SD. *P value <0.05, **P value <0.002, ***P value <0.001, ****P value <0.0001 using two-way ANOVA (*post hoc* Tukey test for multiple comparisons).

Considering the promising results observed during the previous preclinical study conducted in IFNAR(-/-) mice, we evaluated the protective efficacy of the two previous vaccine candidates in sheep, one of the most affected BTV natural hosts. We immunized sheep following a prime-boost strategy consisting of two doses (1x10^8^ PFU) of MVA-VP2-NS1-2A-NS2-Nt or MVA-VP7-NS1-2A-NS2-Nt, administered in a four-week interval. Three weeks after the booster, sheep were subcutaneously challenged with 10^5^ PFU of BTV-4M strain (isolated from sheep blood in KC insect cells and not previously passed through mammalian cell lines, retaining its virulence in sheep) (**Figure 5A**). Thereafter, rectal temperatures, viraemia, RNAemia and hematologic parameters were measured at different days post-infection.

All control sheep developed a steep rise in their rectal temperatures between days 5 and 10 post-infection (**Figure 5B**). Animals immunized with two doses of MVA-VP7-NS1-2A-NS2-Nt also displayed an increase in their rectal temperatures similar to the non-immunized control group (**Figure 5B**). We also observed a strong upsurge in RNAmia in the control group between days 5 and 10 post-infection, coinciding with peak temperature values. RNA levels followed a slow reduction in subsequent days, but RNA was still detected in blood of non-immunized animals at 18 d.p.i. (**Figure 5D**). Infectious virus was detected at high titers in blood of control animals at 5 d.p.i. (mean virus titer = 2,800 PFU/ml) and 7 d.p.i. (mean virus titer = 1950 PFU/ml). Thereafter, although viraemia declined, sheep displayed detectable infectious virus titers in blood at 10 d.p.i. (mean virus titer = 600 PFU/ml) and 12 d.p.i. (mean virus titer = 300 PFU/ml). The RNAmia profile of the MVA/MVA-VP7-NS1-2A-NS2-Nt immunization group was similar to that of the non-immunized control group although mean Ct values were non-significantly lower compared to the control group at days 3, 5, 7 and 10 post-infection (**Figure 5D**). Furthermore, lower levels of infectious virus were measured in blood of these immunized sheep compared the control animals at 5 d.p.i. (mean virus titer = 900 PFU/ml) and 7 d.p.i. (mean virus titer = 550 PFU/ml) (**Figure 5F**). Not just that, animals immunized with the recombinant MVA-VP7-NS1-2A-NS2-Nt did not present infectious virus in blood at 10 and 12 d.p.i., which indicates a faster viral clearance compared to the control group. In addition, it is worth note that we did not detect infectious virus in blood of one MVA/MVA-VP7-NS1-2A-NS2-Nt immunized sheep at any time point after challenge. Besides, this sheep also displayed lower RNA values in blood than non-immunized animals from day 5 post-infection until the end of the experiment. These data demonstrates that prime-boost immunization with MVA/MVAVP7-NS1-2A-NS2-Nt reduces the level and period of viremia in immunized animals after BTV challenge.

In contrast to non-immunized sheep, the MVA/MVA-VP2-NS1-2A-NS2-Nt immunization group presented steady rectal temperatures throughout the experiment, with no increase between 5 and 10 d.p.i. (**Figure 5C**). Indeed, all MVA-VP2-NS1-2A-NS2-Nt immunized animals were aviremic at any day post-challenge as no infectious virus was detected in blood (**Figure 5G**). Moreover, no viral RNA could be detected in blood of these immunized sheep except for day 5 post-infection, when Ct levels of these animals were significantly (P value  <  0.002) lower compared to the control group (MVA/MVA-VP2-NS1-2A-NS2-Nt Ct value mean = 33.515; Control Ct value mean = 26.8975) (**Figure 5E**). Altogether, these results indicate that immunization with the rMVA co-expressing VP2, NS1 and NS2-Nt abrogates viral replication in sheep after BTV challenge.

**Figure 6:**
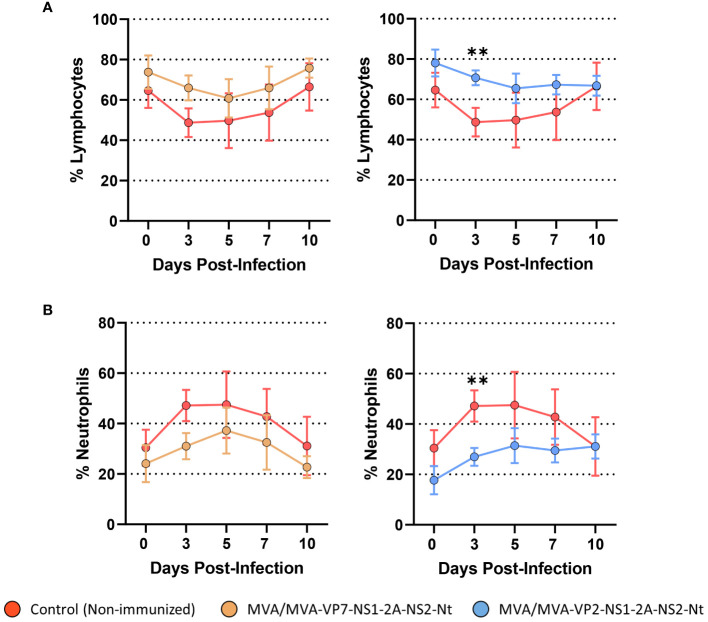
Percentages of lymphocytes and neutrophils in blood from immunized sheep after challenge with BTV-4M. Blood of non-immunized and immunized sheep were analyzed in an autohematology analyzer (BC-5300 Vet; Mindray, China) and the percentage of lymphocytes **(A)** and neutrophils **(B)** based on the total white blood cells were analyzed at days 0, 3, 5, 7, and 10 post-infection. Points indicate the mean value of each group and error bars represent SD. **P value <0.002, using two-way ANOVA (*post hoc* Tukey test for multiple comparisons).

One of the features that characterizes BTV infection is the presence of lymphopenia and neutrophilia in infected animals (44). Non-immunized animals presented these two hematologic features between 3 and 7 d.p.i. followed by the reestablishment of normal percentages of lymphocytes and neutrophils (**Figures 6A, B**). A transient drop in the percentage of lymphocytes as well as a rise in the percentage of neutrophils were also observed in the MVA/MVA-VP7-NS1-2A-NS2-Nt immunized group (**Figures 6A, B**). Nonetheless, these hematologic changes were completely tempered in three out of four MVA/MVA-VP7-NS1-2A-NS2-Nt immunized sheep (including the aviremic sheep) but the remaining sheep suffered lymphopenia and neutrophilia alike non-immunized animals. Immunization with two doses of MVA-VP2-NS1-2A-NS2-Nt prevented animals from developing lymphopenia and neutrophilia at any time point after challenge (**Figures 6A, B**). Overall, this indicates that both vaccine candidates can potentially impair the progression of features that characterizes clinical disease induced by BTV in sheep.

**Figure 7:**
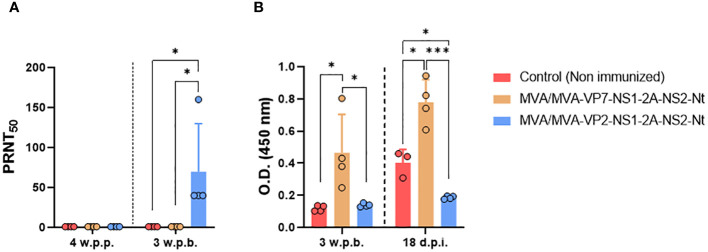
Humoral immune response in immunized sheep after challenge with BTV-4M. **(A)** Neutralizing antibodies titers against BTV-4M in immunized sheep by PRNT_50_ assay. nAbs titers were measured in sera collected at 4 weeks post-prime (w.p.p.), 3 w.p.b. and 18 d.p.i. Bars represent the mean values of each group, points indicate the mean value of each group and error bars represent SD. **(B)** Induction of IgG VP7 antibodies by indirect ELISA in vaccinated animals. Sera dilutions 1:200. Bars represent the mean values of each group and error bars represent SD. Asterisks denote significant differences between groups. *P value <0.05, ***P value <0.001, using two-way ANOVA (*post hoc* Tukey test for multiple comparisons).

We also evaluated the humoral immune response before and after challenge with BTV-4M (**Figure 7**). A homologous neutralizing response was only detected prior to challenge (4 w.p.b.) in sera from sheep immunized with two doses of MVA-VP2-NS1-2A-NS2-Nt, showing BTV-4 nAbs titers ranging between 1:40 and 1:160 (**Figure 7A**). No nAbs against heterologous serotypes (BTV-1 and BTV-8) were detected at 4 w.p.b. (titers below 1:5). We also analyzed VP7 seroconversion by indirect ELISA (**Figure 7B**). As could be expected, we observed that only the MVA/MVA-VP7-NS1-2A-NS2-Nt immunized sheep exhibited antibodies raised against VP7 prior to viral challenge. Unsurprisingly, we registered a boost on IgG titers against VP7 after inoculation with BTV-4M of this immunization group. Neither the non-immunized nor the MVA/MVA-VP2-NS1-2A-NS2-Nt immunization groups developed a humoral response against the protein VP7 before challenge. Nonetheless, sheep immunized with two doses of MVA-VP2-NS1-2A-NS2-Nt did not seroconvert to VP7 after challenge with BTV-4M, which contrasts with the induction of VP7-specific IgG in the non-immunized control group at 18 d.p.i. This supports a robust impairing of viral replication induced by the MVA-VP2-NS1-2A-NS2-Nt vaccine candidate.

## 4 Discussion

Vaccination against BTV is the most appropriate measure for effective control and prevention of BT. Success of vaccination campaigns using conventional vaccine approaches are beyond doubt. A vaccine against BTV-2 was the first inactivated vaccine used in the field after the emergence of BT in Europe (45). Inactivated monovalent vaccine against BTV-4 and bivalent vaccines against BTV-2 and -4 were successfully used in Corsica, Spain, Portugal and Italy (46–48). Nonetheless, important obstacles still exist regarding BTV vaccination, e.g., the inability to distinguish between vaccinated and infected animals (DIVA strategy) and the absence of broad protective immunity against multiple BTV serotypes. In previous works, we described the nonstructural proteins NS1 and NS2-Nt as BTV antigens able to confer multiserotype protection against BTV (27, 28). We also observed that the combined expression of these two BTV proteins induced potent antigen-specific T cell responses that conferred protection against clinical disease in sheep (28). Here, we attempted to strengthen the previously observed cell-mediated immune response against BTV by the simultaneous expression of VP7, NS1 and NS2-Nt. In parallel, we also pursued to confer protection against BTV through the combination of both arms of the adaptive immune response by co-expressing VP2 along with NS1 and NS2-Nt.

The high degree of amino acid sequence conservation of protein VP7 among BTV serotypes justified its targeting for generation of recombinant vaccines (25). In fact, the identity among the VP7 proteins of serotypes 1, 4 and 8 used in this work analyzed with UniProt was between 99.71% and 100%. Besides, major CD4+ and CD8+ T cell epitopes exist within its sequence (49). Here, we recorded the induction of a VP7-specific CD8+ T cell response after immunization with our rMVAs expressing this BTV core surface protein. Nonetheless, this immunogenic response did not correlate with robust protection in mice after a virulent challenge with the homologous BTV-4M. After immunization with the rMVA co-expressing VP7, NS1 and NS2-Nt, results on protection were identical to those of the MVA-NS1-2A-NS2-Nt immunization groups, which implies that the conferred protection would be driven by the expression of the two nonstructural proteins. Nonetheless, immunization with MVA-VP7 did induce a transient blockage of viral replication during initial stages after viral challenge. These results agree with previous works conducted in the IFNAR(-/-) mouse model. Heterologous immunization strategies combining subunit or DNA and MVA-based vaccines expressing VP7 induced an antigen-specific CD8+ T cell response, slowed down disease progression and viral replication at initial stages of infection, but were unable to protect immunized animals against BTV (34, 39, 50–52). Therefore, the role of protein VP7 in protection against BTV seems to be negligible. However, it is important to note that significant protection has been elicited after immunization with a recombinant adenovirus expressing VP7 in IFNAR(-/-) mice (30, 31). Indeed, this is the unique vaccine candidate based on the protein VP7 that has shown some protection against BTV in a natural host (31). Thus, considering data on protection of this adenoviral vaccine candidate in sheep and the existing differences on both the innate and adaptive immune system between mammalian species (53, 54) [and more specifically between mice and ruminant MHC class I and II system (55)] that could sheer vaccine responsiveness of mice and ruminants to a given antigen, we decided to assess the protective efficacy of the MVA-VP7-NS1-2A-NS2-Nt in sheep. This vaccine candidate induced a substantial degree of protection in sheep. Other VP7-based vaccine approaches have provided similar protection against BTV in sheep. For instance, immunization with Core-like particles (CLPs), composed of proteins VP3 and VP7, led to a poor reduction of viral replication and clinical disease (56, 57). Different recombinant viral vectored vaccines based on viruses from the family *Poxviridae*, e.g., capripoxviruses and leporipoxviruses, or the non-replicative canine adenovirus type 2 expressing VP7, might be more comparable examples. Similar to CLPs, they were immunogenic but poorly protective against BTV in natural hosts, as viral replication and disease progression were not hampered (32, 58). Even in the case of the adenoviral vaccine, which showed superior protective response probably due to a higher vector potency and/or host genetics background of the selected sheep breed in relation to disease resistance and vaccine responsiveness (59, 60), viral replication still occurred at high levels although clinical disease was stifled (31). In any case, it seems quite probable that the protection observed in sheep immunized with MVA-VP7-NS1-2A-NS2-Nt was mainly mediated by an immune response specific of the two nonstructural proteins, which eventually concurs with the results observed in mice and most data gathered through the years regarding the formulation of VP7 for vaccine evaluation. Nonetheless, the protective role of the VP7 cell-mediated response in sheep should not be neglected from now on. The immunogenic potential of VP7 could be concealed by the immunogenic response of NS1 and NS2-Nt, whose co-expression may be enough to attain the full protective extent of the cell-mediated protection against BTV. Therefore, it would be desirable to prove the protection induced by VP7 expressed alone or combined with NS1 or NS2-Nt. Regardless, it would be interesting to study whether the inclusion of VP7 in the vaccine composition could influence the type of T cell memory immune response and it improve long-term protection.

The rationale behind the selection of NS1 and NS2-Nt as antigens capable of eliciting multiserotype protective responses relied on their widely shared antigenic determinants among BTV serotypes (28, 61, 62). rMVAs expressing the BTV NS1 protein induced a fully protective CTL response in IFNAR(-/-) mice against different BTV serotypes in absence of nAbs (26). Moreover, this multiserotype protection induced by NS1 can avoid clinical disease development and lessen viral replication in sheep (63). Co-expression of NS2-Nt significantly improves the protection conferred by NS1 in both mice and sheep (28), and that is why we included both antigens in our vaccine formulation. Previously, we cloned genes that encode for these non-structural proteins in individual loci of the rMVA (MVA-NS1-NS2-Nt) (28). Here, we cloned both genes as one, including the peptide 2A into the fusion point, to maximize the cloning capacity of the viral vector used. This strategy may have some implications in terms of immunogenicity and protection. Although we did not make a comparison that could clarify it, the induced NS1 and NS2-Nt-specific CD8+ T cell responses were comparable between MVA-NS1-2A-NS2-Nt and MVA-NS1-NS2-Nt, where the two antigens where cloned in two different loci (28). Regarding protection, a single dose of MVA-NS1-2A-NS2-Nt induced partial protection in mice whereas a single dose of MVA-NS1-NS2-Nt showed a superior protective capacity (28). Since CD8+ T cell epitopes are linear and do not depend on protein conformation (64), the expression of the polyprotein NS1-2A-NS2-Nt should not have implications in this regard. Indeed, expression of this polyprotein by rChAdOx1-NS1-NS2-Nt did not affect the protection elicited against BTV (28). Therefore, it might be due to differences on the cloning site and/or the promoter that controls protein expression. According to our previous work, it seems that protection against BTV hinges on protein NS1 more than NS2-Nt (28). In the case of the MVA-NS1-NS2-Nt, the gene that encodes for the protein NS1 was cloned in the F13L locus under control of a vaccinia virus (VV) Early/Late promoter, whereas the NS1-2A-NS2-Nt gene was cloned in the TK locus of the rMVA under control of the VV Early/Late p7.5 promoter. Previously, we observed that cloning of a given BTV antigen in the F13L locus under control of a VV Early/Late promoter resulted in a superior immunogenic and protective response compared to cloning in the TK locus under control of the VV Early/Late p7.5 promoter (unpublished data). Therefore, it could explain the observed differences. In any case, these differences on protection were absent when we applied a prime-boost regimen with MVA-NS1-2A-NS2-Nt and, more importantly, the multiserotype capability of these antigens was preserved. Besides, neither the co-expression of VP2 or VP7 along with NS1 and NS2-Nt induced interferences of any kind regarding immunogenicity, as nAbs titers and antigen-specific CTLs responses were identical to those induced by the rMVAs individually expressing these antigens.

In sheep, we observed a partially protective response against BTV after immunization with MVA-VP7-NS1-2A-NS2-Nt. As stated previously, co-expression of NS1 and NS2-Nt likely determines this partial protection. It is worth note that the heterologous combination of ChAdOx1/MVA co-expressing NS1 and NS2-Nt significantly diminished viral replication after peak viraemia levels (28). Here, we observed that the protection mediated by NS1 and NS2-Nt also reduced the presence of infectious virus and viral RNA in blood, although it seems that the heterologous prime-boost strategy is superior in terms of protection. In this regard, homologous prime-boost strategies show less efficiency to stimulate cell-mediated immune responses than heterologous regimes (65). Humoral immunity is less affected by utilization of same recombinant vectors (65), which explains why the nAbs response induced by our rMVAs is robust whereas the cell-mediated response is slightly dampened compared to the heterologous combination. Also, a BTV-induced acute inhibition of CD8+ T cell activation at the peak of BTV replication might affect the recall of cytotoxic responses induced by the vaccine (66).

The combination of nAbs and CTLs is crucial for the development of long lasting immunity against BTV (62, 67) so that an effective vaccine should aim to induce both. To do so, we designed a rMVA that co-expressed VP2, the major inductor of nAbs, and NS1 and NS2-Nt, inducers of durable and cross-protective CD8+ T cell immunity to BTV (27, 28). Importantly, we did not found interferences between VP2, NS1 and NS2-Nt in terms of immunogenicity or protection against homologous and heterologous BTV serotypes as stated above. The efficacy of a vaccine against arboviruses is divided into protection against disease and blocking of onward virus transmission to the insect vector (68). On the one hand, MVA-VP2-NS1-2A-NS2-Nt immunization prevented sheep from developing clinical disease after virulent challenge with the homologous BTV-4M. On the other hand, virological parameters reflected a robust protection induced by this recombinant vaccine candidate. Viraemia was absent in the immunized sheep and just nearly undetectable levels of RNA were detected at day 5 post-infection. Amongst the two assays, detection of viral RNA by RT-qPCR shows a higher sensitivity but cell culture isolation is the method that demonstrates the presence of infectious virus. Thus, we can affirm that our vaccine candidate expressing VP2, NS1 and NS2-Nt provides strong protection against BTV in sheep, one of the most affected hosts. The absence of seroconversion to VP7 also supports it, as this likely relates to a hindering of viral replication since initial stages after challenge. A complete blockage of viral replication in immunized animals would undoubtedly impair transmission to Culicoides insect vectors. Furthermore, if we consider that infectious virus titers in systemic blood exceeds those found in skin (69), and the fact that Culicoides infection is dose-dependent, with the 50% midge alimentary infective dose (MAID_50_) estimated to a blood meal titer between 10^5^ and 10^6^ TCID50/mL (70–72), we can affirm that this vaccine candidate potentially grants a full protection against BTV that would break transmission cycle.

Inactivated BT vaccines are licensed in Europe for different serotypes and have significantly contributed to a reduced circulation and eradication of the virus (73, 74). However, they are serotype specific [although some mild cross-protective responses can be induced (75, 76)] and less immunogenic than LAVs. Besides their superior immunogenicity, LAVs are more likely to induce cross-protective responses than inactivated vaccines (68), which may lie on presentation of conserved antigens that stimulates cross-reactive CTL responses. Nevertheless, under attenuation, onwards transmission and significant side effects in immunized animals have made the implementation of LAVs difficult. Alternative LAVs approaches that partly solves inherent LAV safety concerns have been studied, e.g. Disabled Infectious Single Animal (DISA) and Disabled Infectious Single Cycle (DISC) (77, 78). Recently, Van Rijn, P. and colleagues developed a pentavalent DISA vaccine that solves major safety drawbacks of LAVs while maintaining a high immunogenic profile (79). In this work, authors presented a multivalent approach that conferred complete protection in cattle and very significant protection in sheep against serotypes 2 and 8 of BTV. Importantly, although not all immunized sheep displayed a neutralizing response against BTV-2 after immunization three out of four of these animals were protected, which indicates that cell-mediated immunity plays an important role in protection. Cocktails of DISC vaccines offered similar results in cattle and sheep (80). In essence, these experimental vaccines share the immunological basis of our vaccine candidate MVA-VP2-NS1-2A-NS2-Nt as both arms of the adaptive immune response are stimulated. Furthermore, it is worth note that replication-deficient viral vectors, like MVA, are the most potent approaches in priming T-cell responses to a recombinant antigen (81). Moreover, we formulated in our vaccine two antigens targeted by cytotoxic CD8+ T cells to reduce the likelihood of immune escape. Not just that, these multivalent approaches are dependent on the recovery and stability *in vitro* of all vaccine components (79), which eventually can constrain its multiserotype potential. On the contrary, we present a single and stable recombinant virus with capacity of adequately ensuring humoral and CTL responses that protect against different BTV serotypes.

Multivalent approaches exploiting inactivated or newly generated vaccines based on the induction of a VP2-specific neutralizing response share some potential hindrances that could restrict their prospective implementation. First, immune interferences between different antigens present within the same vaccine formulation have been reported for some viral diseases (82). It is quite probable that particular serotypes of BTV exhibit immunodominance on others, as it occurs with Dengue virus (82). Negative interference was observed during evaluation of a bivalent vaccine based on VLPs of serotype 1 and 4 of BTV (83). Also, after combination of VLPs of serotype 2 and 4, the BTV-2 component elicited a stronger immune response in terms of nAbs (84). Second, implementation of multivalent BT vaccines could be restrained by the mechanism described as ‘antibody-dependent enhancement of infection’ (ADE) (85). ADE has been linked with other RNA viruses (86, 87). In the case of BTV, Attoui, H. et al, described an ADE-like mechanism after inoculation with a non-pathogenic BTV-1 of IFNAR(-/-) mice immunized with recombinant VP2 protein of serotypes 4 and 8 (88). Although no extensive data regarding this issue exist, some ADE of BTV infection can be suggested in calves vaccinated against BTV-8 and challenged with BTV-9 (75). Third, the chance of a negative immune interference in the neutralizing response after a previous encounter with BTV or vaccination due to the presence of cross-reactive non-neutralizing epitopes within the sequence of the protein VP2 is an issue that still need to be addressed. There are several examples in which cross-reactivity impairs the magnitude and duration of subsequent antibody responses. For example, Aydillo, T., and colleagues described the immunological imprinting of the antibody response in COVID-19 patients that encountered seasonal coronavirus prior to SARS-CoV-2 infection, showing that the antibody response to SARS-CoV-2 spike protein is biased by pre-existing immunity against conserved epitopes shared by seasonal betacoronaviruses, hindering the induction of SARS-CoV-2 nAbs directed against novel antigenic epitopes (89). This feature has been also observed among different alphaviruses in equines and humans during vaccine clinical evaluation (90, 91) and other RNA viruses such as Dengue (92) or Influenza (93). Therefore, previous recognition of non-neutralizing cross-reactive VP2 epitopes might steer the immune system by recall of pre-existing memory B cells rather than stimulating *de novo* humoral responses against antigenically important and variable epitopes. The only way to overcome these three potential issues is the formulation of antigens capable of inducing potent multiserotype immune responses against BTV. Our rMVA co-expressing VP2, NS1 and NS2-Nt is a vaccine candidate that would solve all these three potential drawbacks, as not only a serotype-specific neutralizing response mediates protection, but also long-lasting cross-reactive and protective T-cell-mediated responses are induced by the highly conserved NS1 and NS2-Nt proteins.

In summary, this study presents a promising recombinant vaccine candidate against BTV based on the combination of VP2 with NS1 and NS2-Nt. A homologous prime-boost immunization induced a potent immune response that conferred full protection against BTV infection. Moreover, this safe and adjuvant-free vaccine candidate can confer protection against multiple BTV serotypes, and, more importantly, is compatible with a DIVA strategy.

## Data availability statement

The original contributions presented in the study are included in the article/**Supplementary Material**. Further inquiries can be directed to the corresponding author.

## Ethics statement

The animal study was approved by Ethical Review Committee at the INIA-CISA and Comunidad de Madrid (Permit number: PROEX 060.7/21). The study was conducted in accordance with the local legislation and institutional requirements.

## Author contributions

LJ: Conceptualization, Data curation, Formal Analysis, Investigation, Methodology, Software, Writing – original draft, Writing – review & editing. SU: Formal Analysis, Investigation, Methodology, Writing – review & editing. EC: Methodology, Supervision, Writing – review & editing. GL: Methodology, Writing – review & editing. MI: Conceptualization, Methodology, Writing – review & editing. JB: Methodology, Supervision, Writing – review & editing. SM: Methodology, Writing – review & editing. AM: Writing – review & editing. AN: Funding acquisition, Writing – review & editing. JO: Conceptualization, Funding acquisition, Investigation, Project administration, Resources, Supervision, Writing – review & editing.
