# Supplementary material for: Co-expression of VP2, NS1 and NS2-Nt proteins by an MVA viral vector induces complete protection against bluetongue virus
Source: Front Immunol. 2024 Jul 12;15:1440407. doi: 10.3389/fimmu.2024.1440407 (PMC11272488; doi:10.3389/fimmu.2024.1440407)
Supplement: Supplementary file 1 [file DataSheet_1.pdf]

Supplementary Figure 1

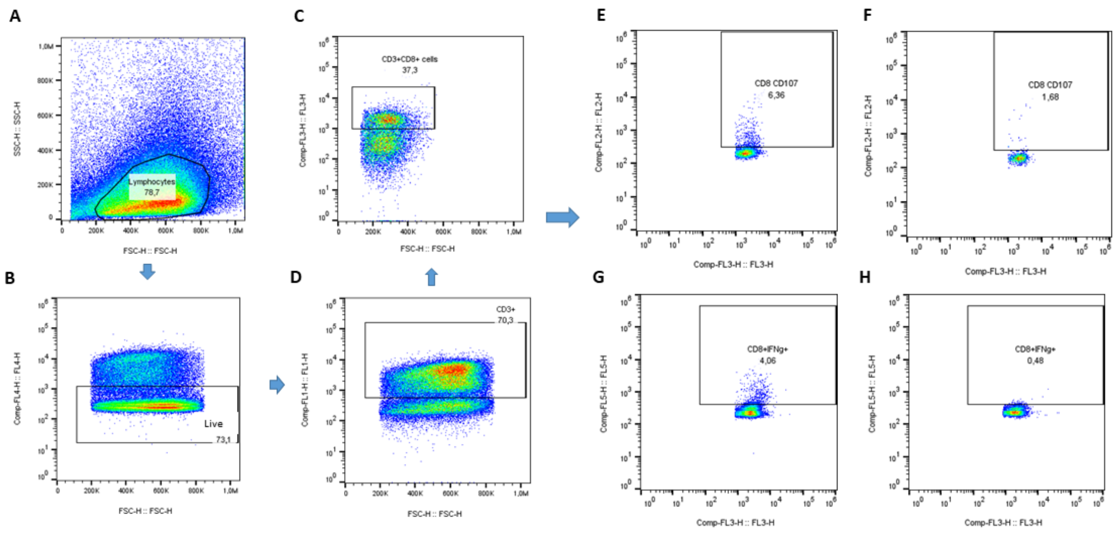

Figure. Gating strategy used to analyze BTV specific T-cell responses. A) Lymphocytes were gated by SSC vs FCS density plot. B) Live lymphocytes were selected by negative zombie staining and (C) T cells were determined as CD3+ positive cells. T CD8+ cells (D) were analyzed for expression of CD107a or IFN-γ. (E-H) Representative dot plots showing specific % CD8+CD107a or %CD8+IFN-γ+ after peptide (152 BTV NS1) stimulation of lymphocytes from vaccinated MVA-NS1-2A-NS2-Nt (E, G) or non-immunized mice (F, H).
